# Serum differential proteomic profiling of patients with isolated methylmalonic acidemia by iTRAQ

**DOI:** 10.3389/fgene.2022.765637

**Published:** 2022-08-29

**Authors:** Sitao Li, Congcong Shi, Yao Cai, Xia Gu, Hui Xiong, Xiaoyu Liu, Yinchun Zhang, Xin Xiao, Fei Ma, Hu Hao

**Affiliations:** ^1^ Department of Pediatrics, The Sixth Affiliated Hospital, Sun Yat sen University, Guangzhou, China; ^2^ Inborn Errors of Metabolism Laboratory, The Sixth Affiliated Hospital, Sun Yat sen University, Guangzhou, China; ^3^ Department of Child Care, The Maternity and Child Health Care Hospital of Tianhe District, Guangzhou, China

**Keywords:** methylmalonic acidemia, iTRAQ, differential proteome, cholesterol metabolism, immunoglobulins, methylmalonyl coenzyme A mutase

## Abstract

Isolated methylmalonic acidemia (MMA) is an inherited organic acid metabolic disorder in an autosomal recessive manner, caused by mutations in the methylmalonyl coenzyme A mutase gene, and the isolated MMA patients often suffer from multi-organ damage. The present study aimed to profile the differential proteome of serum between isolated MAA patients and healthy control. The *in vivo* proteome of isolated MAA patients and healthy subjects was detected by an isobaric tag for relative and absolute quantitation (iTRAQ). A total of 94 differentially expressed proteins (DEPs) were identified between MMA patients and healthy control, including 58 upregulated and 36 downregulated DEPs in MMA patients. Among them, the most significantly upregulated proteins were CRP and immunoglobulins, and the top five most significantly downregulated proteins were all different types of immunoglobulins in MMA patients. GO analysis showed that these DEPs were mainly enriched in immune-related function and membrane protein-related function. KEGG revealed that these DEPs were mainly enriched in lysosome and cholesterol metabolism pathways. Also, these DEPs were predicted to contribute to lipid metabolic diseases. We addressed the proteomes of isolated MMA patients and identified DEPs. Our study expands our current understanding of MMA, and the DEPs could be valuable for designing alternative therapies to alleviate MMA symptoms.

## Introduction

Methylmalonic acidemia (MMA) is an inherited organic acid metabolic disorder in an autosomal recessive manner. According to the biochemical manifestation, MMA can be classified into two common types: isolated MMA and MMA combined with homocysteinemia (Manoli and Venditti, 2005). Isolated MMA is mostly caused by mutations in the methylmalonyl coenzyme A mutase (MCM) gene (*MUT*) (Han et al., 2017), and a few cases have been shown to be the result of other gene mutations, such as *MMAA*, *MCEE*, and others (He et al., 2020). MMA usually resulted in various clinical symptoms, including severe metabolic acidosis, thrombocytopenia, hyperammonemia, ketosis and ketonuria, developmental delay, neutropenia, and hyperglycinemia (Manoli and Venditti, 2005). Clinical diagnosis of MMA relies upon specialized metabolic testing. Definitive diagnosis of MMA relies on the analysis of organic acids in plasma and/or urine by gas-liquid chromatography and mass spectrometry; the concentration of methylmalonic acid is greatly increased in the plasma, urine, and cerebrospinal fluid of affected individuals (Manoli et al., 1993). Based on complex and comprehensive clinical diagnosis, the abnormal production of metabolites in MMA patients has been relatively clear, but proteomic changes in MMA patients are rarely reported.

Isolated MMA patients with mutations in the *MUT* gene are usually severely ill with poor prognosis, high early mortality, and high lifelong morbidity (Jiang et al., 2020; Liang et al., 2021). The age of death in children with isolated MMA was 2 years, ranging from 5 days to 15 years, and 40% of individuals died and the overall mortality rate was 36%, with all deaths occurring during or after the acute metabolic crisis (Dionisi-Vici et al., 2006). The primary treatment for patients with isolated MMA is dietary restriction of propyl amino acid and carnitine supplementation. Despite treatment, the metabolite profile of the patients with isolated MMA remains abnormal, and the prognosis is still poor. Although several hypotheses have been proposed, the pathologic mechanism of abnormal protein expression in progressive isolated MMA systemic injury remains to be elucidated.

Recently, the characterization of MMA with homocystinuria and cobalamin deficiency type C (cblC) proteome in fibroblasts found that the downregulated proteins were mainly enriched in cellular detoxification (Hannibal et al., 2011). Also, the proteome in circulating lymphocytes in cblC patients has been demonstrated (Caterino et al., 2015). However, the proteomic characteristics of isolated MMA patients are still unclear. The present study investigated the proteome of serum isolated from three isolated MMA patients with *MUT* treated with a multi-drug combination. These three patients still have abnormal levels of metabolites although receiving treatment. This study of the proteome in isolated MMA patients was performed using the isobaric tag for relative and absolute quantitation (iTRAQ) analysis. Proteome identification helped bridge the gap between the role of *MUT* gene products and the clinical manifestations of MMA defects in humans.

## Materials and methods

### Ethics statement

This project was approved by the Ethical Committee of The Sixth Affiliated Hospital of Sun Yat-sen University and adhered to the World Medical Association’s Declaration of Helsinki (WMADH 2008). In this study, MMA analysis was performed with written informed consent from guardians of each pediatric patient, following the approval of The Sixth Affiliated Hospital of Sun Yat-sen University.

### Patient selection

The serum for three MMA patients and three healthy subjects from The Sixth Affiliated Hospital of Sun Yat-sen University was collected. Genotypes of MMA donors are listed in Table 1; the virulence gene of three MMA patients is *mut* gene. Clinical diagnosis of MMA patients relies upon specialized metabolic testing, which is performed by the Genetic Metabolic Disease Detection Laboratory of the Sixth Affiliated Hospital of Sun Yat-sen University. Table 2 listed the detailed clinical features of MMA patients determined by gas–liquid chromatography and mass spectrometry and also listed the detail of laboratory biochemical parameters. Serum from normal control and patients with confirmed MMA were subjected to iTRAQ assay for investigating the differentially expressed proteins (DEPs). Moreover, to verify the clinical characteristics of candidate DEPs, we subsequently collected the clinical biochemical data from 14 MMA patients and 15 non-MMA and uninfected patients. These 29 samples were in addition to those analyzed in the iTRAQ experiment. The information of these 29 samples is shown in Supplementary Table S1. All frozen tissue samples in liquid nitrogen were stored at −80°C.

**TABLE 1:** List of MMA donors with their genotypes and clinical features used in this study.

Sample ID	Age	Virulence gene	Genetic mutation	Medication before blood collection
001	16 months	Sequence alterations in *MUT*	c.636G>A homozygous	Tacrolimus, sesepin, lapindine, and zorcarnitine
002	10 months	Sequence alterations in *MUT*	c.323G>A, c.914 T>C compound heterozygote	Arginine, vitamin B_12_, levocarnitine, and glutathione
003	1.5 months	Sequence alterations in *MUT*	c.755dupA, c.1159 A>C	Arginine, vitamin B_12_, and levocarnitine
004	3 years	Healthy control	-	-
005	6 years	Healthy control	-	-
006	4 years	Healthy control	-	-

**TABLE 2:** Clinical information of MMA patients prior to sampling.

Biochemical	Sample 001	Sample 002	Sample 003
Body weight (kg)	8.5 kg	8 kg	3.44 kg
Height (cm)	80 cm	72 cm	54 cm
Malnutrition	Moderately nourished	Moderately nourished	Malnutrition: moderate
pH	7.353	7.47	7.38
PCO_2_ (mmHg)	25.9	16.9	24
PO_2_ (mmHg)	112.7	121.2	69
HCO_3_ (mmol/L)	12.8	8.38	14.2
Lac (mmol/L)	3.1	2.9	6.6
Gap (mmol/L)	17.4	21.9	19.4
Ammonia (μmol/L)	176–84.5	149–66.9	170–67.6
CRP (mg/L)	0.91	<0.50	<0.50
White blood cell	5.97 × 10E9/L	6.58*10E9/L	7.75 × 10E9/L
Red blood cell	3.13 × 10E12/L	4.39*10E12/L	3.07 × 10E12/L
NEUTR	0.259	0.211	0.196
NLR	0.58	0.61	0.690
HGB (g/L)	89	99	87
PLT	228 × 10E9/L	253*10E9/L	758 × 10E9/L
Urine MMA (μmol/L)	964.8	1077.4	1278
C3 (μmol/L)	24.65	55.22	16.78
C0 (μmol/L)	32.56	76.13	22
C2 (μmol/L)	27.23	48.26	14.66
C3/C0 (0.01–0.25)	0.76	0.73	0.76
C3/C2 (0.01–0.25)	0.91	1.14	1.14
C3/C16 (0.15–2.0)	25.41	26.05	31.1
Glycine (μmol/L)	281.20	221.55	354.6
Valine (μmol/L)	20.97	88.53	116.65

### Protein extraction and isobaric tag for relative and absolute quantitation labeling

Frozen serum samples were extracted by using RIPA lysis buffer (Elabscience, China) and subsequently homogenized by sonication (Scientz) on ice. The homogenate was cleared using centrifugation at 12,000 rpm for 15 min at 4°C. The major proteins of albumin and IgG were removed using ProteoExtract™ Albumin Removal Kit (Cat. No.:122640, MERCK). After that, the protein concentrations of supernatants were determined by Pierce BCA Protein Assay Kit. Subsequently, 20 μg of each sample was taken and tested by SDS-PAGE. The qualified protein sample entered the iTRAQ labeling stage. Total protein (150 μg) from each sample solution was mixed with 200 μl of 8 mol/L urea in Nanosep Centrifugal Devices (PALL, United Kingdom) and centrifuged at 14,000 g at 20°C for 20 min. All following centrifugation steps were performed applying the same conditions allowing maximal concentration. Samples were incubated in 20 μl of 50 mmol/L iodoacetamide for 30 min in the dark to block reduced cysteine residues followed by centrifugation, and the liquid at the bottom was discarded. The Tris–HCl (pH 8.0) was added to every tube, centrifuged at 12,000 rpm for 10 min, and the solution was discarded at the bottom. Then, 200 μl of 100 mmol/L triethylammonium bicarbonate (TEAB) buffer was added to tubes and centrifuged twice. The solution was subjected to proteolytic digestion (1:50) overnight at 37°C. The digests of peptides were collected by centrifugation following iTRAQ labeling.

Each iTRAQ-label reagent (AB Sciex, United States) was dissolved in 70 μl of isopropanol. The digested-peptide samples were dissolved in 30 μl TEAB buffer (200 mM). Next, the iTRAQ-label reagent was added to the respective peptide mixture for 2 h. The labeling reaction was quenched by the addition of 100 μl of MilliQ water, and six labeled samples were then pooled into one sample according to the manufacturer’s instructions. After pooling, the samples were evaporated by vacuum concentration to remove excess water, TEAB, and isopropanol.

### One-dimensional high-pH reversed-phase chromatography separation

The freeze-dried samples were dissolved in 100 μl of mobile phase A (10 nM ammonium formate, 5% acetonitrile, pH 10.0). Peptide separation was performed on a Thermo UltiMate 3000 UHPLC, and the chromatographic column was purchased from Agilent (ZORBAX Extended-C18, 2.1). The detection wavelength was UV 215 nm, and the flow rate was 0.3 ml/min. Mobile phase B (10 nM ammonium formate, 90% acetonitrile, and pH 10.0) separation gradient was linear from 5 to 38% in 80 min. One tube was collected every 1 min within the gradient range, and a total of 16 tubes of elution solution were collected, centrifuged, and dried for LC-MS analysis.

### Liquid chromatography tandem mass spectrometry analysis and data reorganization

The LC-MS/MS analysis was conducted by Wininnovate Bio (China). Briefly, the lyophilized peptide fractions were re-suspended in 0.1% formic acid and then loaded into a nanoViper C18 (3 μm, 100Å) trap column, and online chromatography separation was performed on the Easy nLC 1200 system (ThermoFisher, United States). The trapping and desalting procedure was carried out with a volume of 20 μl 100% solvent A (0.1% formic acid). Then, an elution gradient of 5–38% solvent B (80% acetonitrile, 0.1% formic acid) at a flow rate of 300 nl/min (0–50 min, 5–38% B; 60–60 min, 38–100% B) in 60 min was used on an analytical column (50 μm × 15 cm C18-3 μm 100 Å). The data-dependent acquisition (DDA) mass spectrum techniques were used to acquire tandem mass spectrometry (MS) data on a ThermoFisher Q Exactive mass spectrometer (ThermoFisher, United States) fitted with a Nano Flex ion source. Data were acquired using an ion spray voltage of 1.9 kV, and an interface heater temperature of 275°C. The MS was operated with FULL-MS scans. For DDA, survey scans were acquired in 250 ms and up to 20 product ion scans (50 ms) were collected. Normalized collision energy (NCE) was set to 30 eV. Only spectra with a charge state of 2–4 were selected for fragmentation by higher-energy collision energy. Dynamic exclusion was set for 25 s. The proteomic raw data have been deposited on PRIDE Archive (https://www.ebi.ac.uk/pride/archive) with the project accession: PXD034075.

### Data reorganization

The MS/MS data were analyzed for protein identification and quantification using the Proteome discoverer (v2.1.0.81). The local false discovery rate was 1.0% after searching against the Human protein database with a maximum of two missed cleavages and one missed termini cleavage. The following settings were selected: oxidation (M), acetylation (Protein N-term), deamidation (NQ), Pyro-glu from E, Pyro-glu from Q for variable modifications as well as carbamidomethylation (C), iTRAQ8plex (N-term), and iTRAQ8plex (K) for fixed modifications. Precursor and fragment mass tolerance were set to 10 ppm and 0.05 Da, respectively. A full list of proteins identified and relatively quantified by iTRAQ is shown in Supplementary Table S4. A full list of peptide groups identified and relatively quantified by iTRAQ is shown in Supplementary Table S5.

### Statistical analysis of isobaric tags for relative and absolute quantitation data

DEPs were screened by t-test of the “*limma*” R package, and proteins with *p*-value < 0.05 and fold change (FC) ≥ 1.2 were considered DEPs.

### Functional annotation

For functional annotation, the proteomic data were normalized, and then the protein expression of each sample was analyzed for differences to screen the DEPs using a t-test provided by the “*limma*” R package. The screening criterion of DEPs was that fold change (FC) ≥ 1.2 and the *p*-value < 0.05, between the two groups. Next, cluster analysis, principal component analysis (PCA), and partial least squares discrimination analysis (PLS-DA) were performed on DEPs, and they were referred to the Gene Ontology (GO) and Kyoto Encyclopedia of Genes and Genomes (KEGG) database, among which the “*ggplot2*” R package was used for volcano plots, heatmap, and PCA. The “*ropls*” R package was used for PLS-DA. For gene set enrichment analysis (GSEA), the whole genome was ranked from the largest to smallest based on the logFC of DEPs and matched to the KEGG pathway database, to draw a GSEA plot. A protein–protein interaction (PPI) network was constructed by Cytoscape version 3.6.1 using DEPs.

## Results

### Quantitative proteomic profiling of serum of patients with isolated methylmalonic acidemia by isobaric tags for relative and absolute quantitation labeling

**FIGURE 1:**
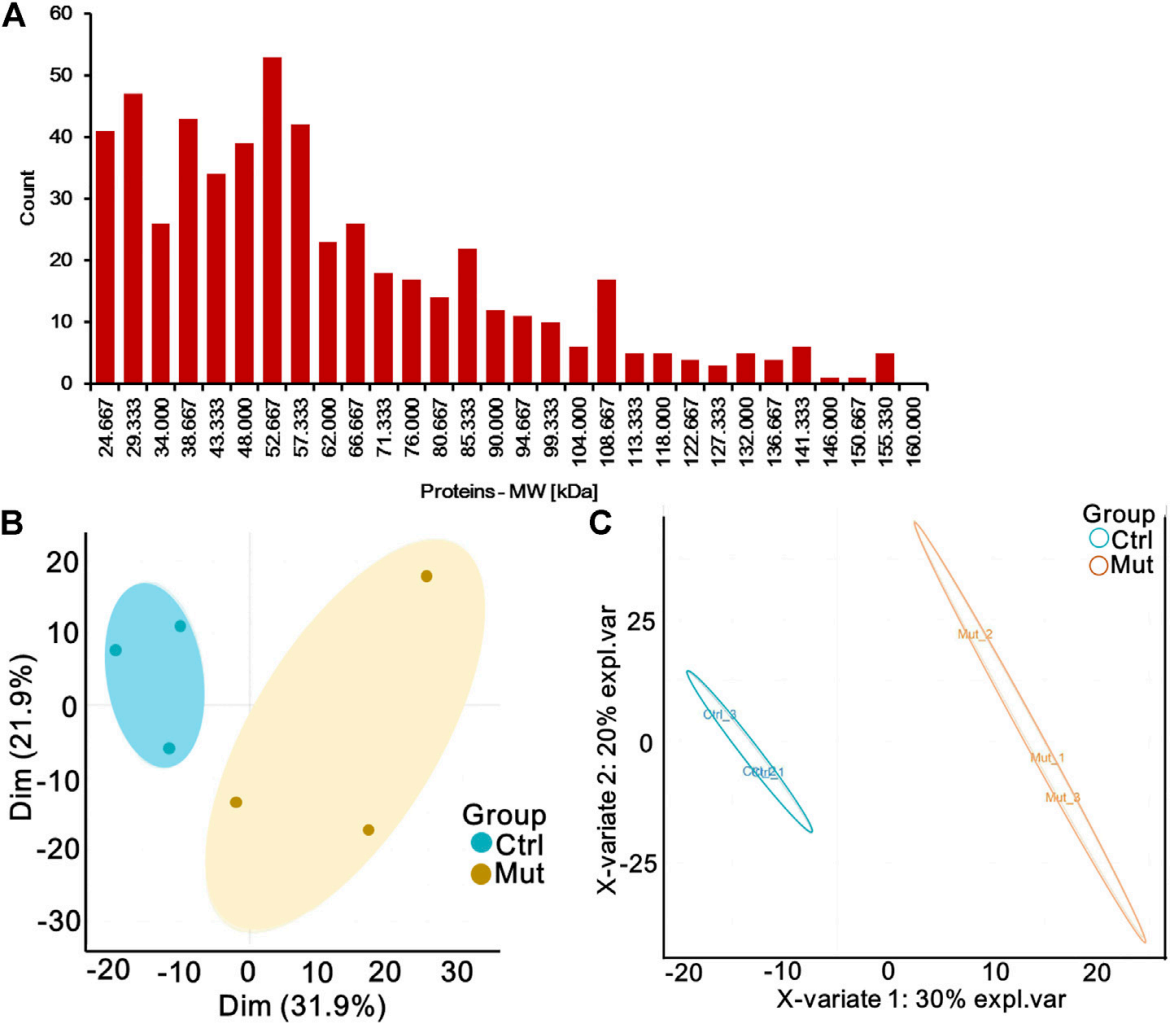
Quantitative proteomic profiling of serum of patients with MMA by iTRAQ labeling. **(A)** Histogram of the distribution of the average molecular weight of protein. **(B)** Principal component analysis (PCA) diagram of six samples. Oval indicates the 95% confidence intervals. **(C)** Partial least-squares discriminant analysis (PLS-DA) diagram of six samples based on all proteins. Oval indicates the 95% confidence intervals.

Serum of patients with isolated MMA and healthy donators was subjected to proteomic analysis to elucidate proteomic changes. A total of 10306 peptides were identified, of which 9722 were uniquely mapped to known sequences of our iTRAQ proteomic results. Moreover, we identified 770 proteins whose average molecular weight was between 24 and 80 kDa (Figure 1A). PCA and PLS-DA analysis showed an obvious separation in the control group and mut group with the 95% confidence intervals, implicating that proteins were significantly separated (Figures 1B,C). Therefore, these data were used for further analysis.

### Differential proteomics analysis

**FIGURE 2:**
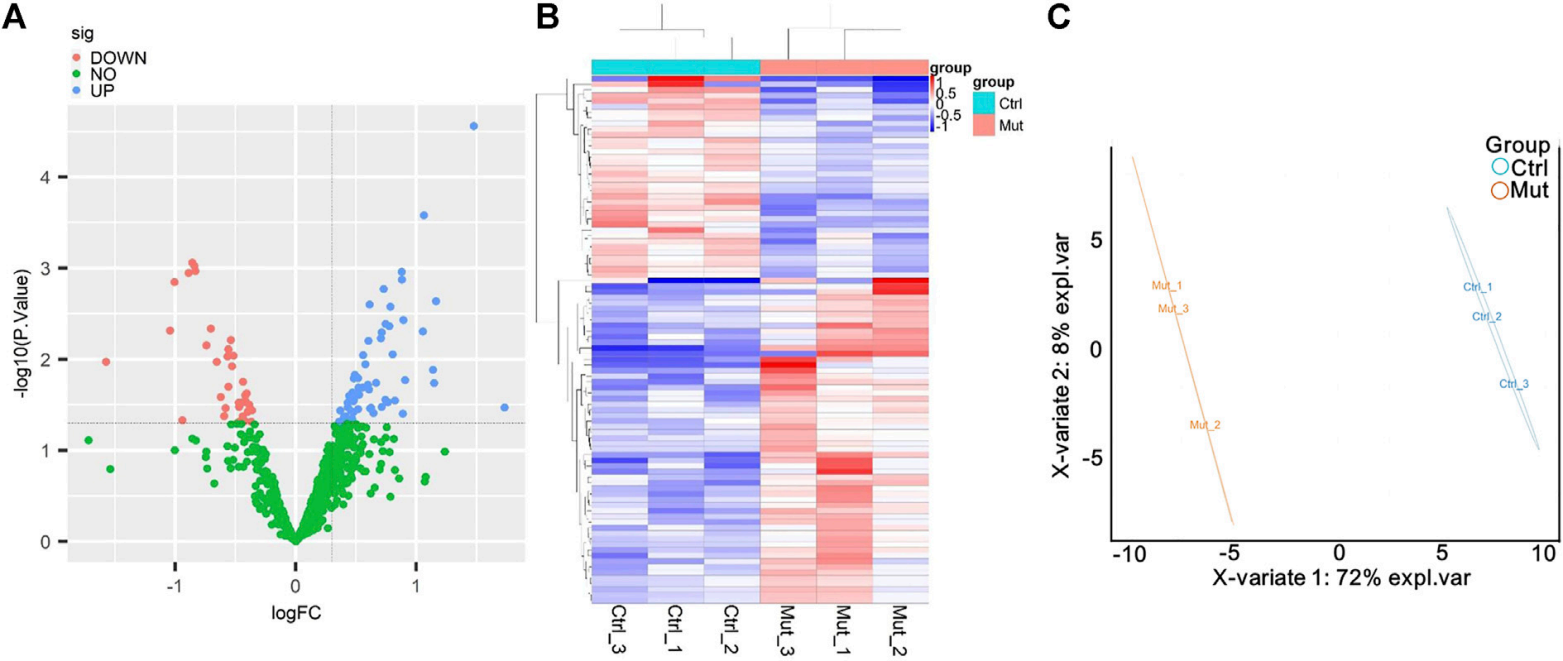
Analysis of the differentially expressed proteins (DEPs) between the MMA patients and healthy control. **(A)** Volcano plot of DEPs. The blue dot represents DEPs upregulated, and the red dot represents DEPs downregulated in the MMA patients compared with the healthy control. **(B)** Cluster heatmaps of significant DEPs. MUT represents the serum that was obtained from the MMA patients, and Ctrl represents the serum obtained from the healthy control. Each column represents a sample, and the row shaded in red represents upregulated, while shaded in blue represents downregulated when MMA patients vs. healthy control. **(C)** PL-SDA diagram of six samples based on DEPs. Oval indicates the 95% confidence intervals.

To search for MMA-induced specific proteins, proteome differential analysis was performed. As shown in the volcano plot, MMA induction upregulated the expression of 58 proteins and downregulated the expression of 36 proteins (Figure 2A). MMA and control groups were analyzed by the heatmap overview showing a good separation between the two groups, as well as confirmed by the PLSDA (Figures 2B,C). All the details of DEPs are listed in Supplementary Table S2, compared with the control group, the most significantly upregulated proteins were C-reactive protein (CRP), immunoglobulin, collagen alpha-2 (XI) chain (COL11A2), GTP-binding protein SAR1a (SAR1A), and dipeptidyl peptidase 1 (CTSC), and the top five most significantly downregulated proteins were all immunoglobulins, followed by apolipoprotein (APO), tubulin beta chain (TUBB), and cholesteryl ester transfer protein (CETP).

### Functional analysis of differentially expressed proteins

**FIGURE 3:**
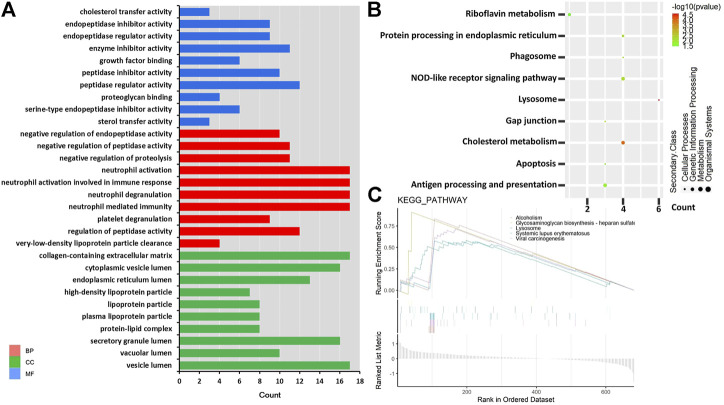
GO and KEGG pathway enrichment based on DEPs. **(A)** Top 30 GO terms in three domains. Red shows DEPs enriched in the biological process (BP); blue shows DEPs enriched in the molecular function (MF); and green shows DEPs enriched in the cellular component (CC). The left is the name of the GO term, while the right column represents the counts of DEPs. **(B)** KEGG enrichment items. The left is the name of the KEGG pathway, the right represents enrichment, and the size of the solid circle indicates the number of genes. **(C)** GSEA of identified DEPs. The abscissa is the list of whole genes sorted by logFC; the upper half of the ordinate is divided into enrichment scores, the lower half is divided into log2FC, and the pathway type is represented by the color label.

To further explore the potential functions of DEPs, GO, and KEGG predictions were performed. GO functional analysis showed that DEPs were mainly related to immune function (such as neutrophil-mediated immunity, neutrophil activation, neutrophil degranulation, neutrophil activation involved in immune response) and membrane protein function (such as vesicle lumen, cytoplasmic vesicle lumen, collagen-containing extracellular matrix) (Figure 3A). The KEGG pathway analysis showed that six DEPs were significantly enriched in the lysosome, including CTSC, tartrate-resistant acid phosphatase type 5 (ACP5), lysosomal protective protein (CTSA), cathepsin B (CTSB), prosaposin (PSAP), and tripeptidyl-peptidase 1 (TPP1), these six DEPs were downregulated in MMA patients (Figure 3B, Supplementary Figure S1). Also, four DEPs were significantly enriched in cholesterol metabolism, including APOE, APOC2, APOB, CETP, and these four DEPs were downregulated in MMA patients (Figure 3B, Supplementary Figure S2). We also found that the classical immune pathway of the NOD-like receptor pathway was also significantly enriched by these DEPs. Moreover, we used GSEA to further explore the potential function of DEPs, consistent with the KEGG results, the lysosome pathway was also observed in the GSEA-plot with the top five significant enrichment pathways, as well as an autoimmune disease pathway of systemic lupus erythematosus (Figure 3C). These results suggested that the disease progression of MMA may be involved not only in immune disorders but also related to abnormal lysosomal function and cholesterol metabolism.

### Differentially expressed proteins associated with lipid metabolic diseases

**FIGURE 4:**
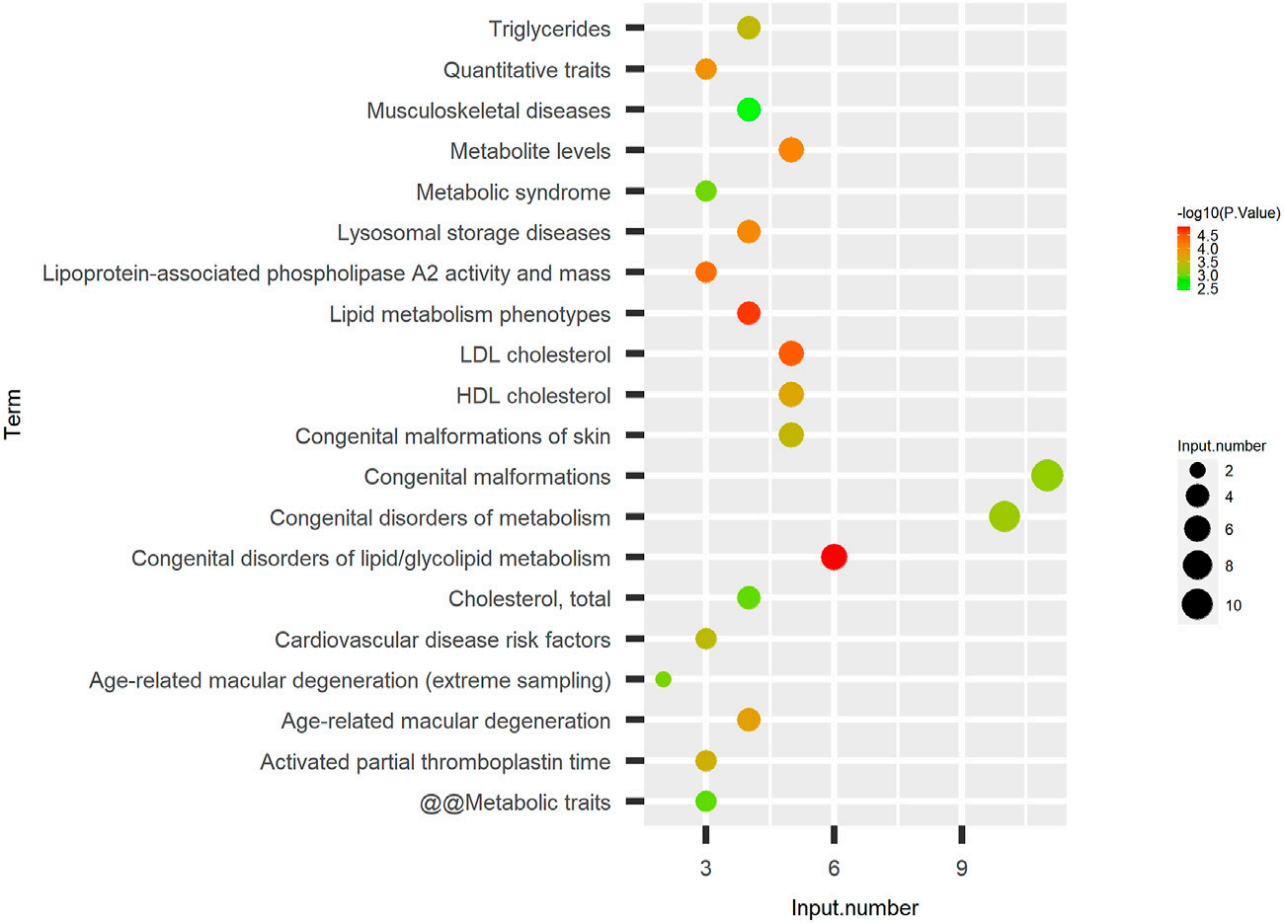
DEPs associated with lipid metabolic diseases. Disease enrichment analysis of identified DEPs. The left is the name of the disease, the right represents enrichment, and the size of the solid circle indicates the number of genes; the color of the solid circle indicates the–log10 (*p*-value) of enrichment.

Furthermore, we predicted the relationship between DEPs and human disease. As shown in Figure 4, the diseases most significantly associated with these DEPs were as follows: congenital disorders of lipid/glycolipid metabolism, lipid metabolism phenotypes, LDL cholesterol, lipoprotein-associated phospholipase A2 activity and mass, and HDL cholesterol. In addition, other diseases related to lipid metabolism were also predicted, such as triglycerides and cholesterol total. These results imply that DEPs are widely involved in lipid-abnormal metabolic diseases.

### Protein–protein interaction network analysis

**FIGURE 5:**
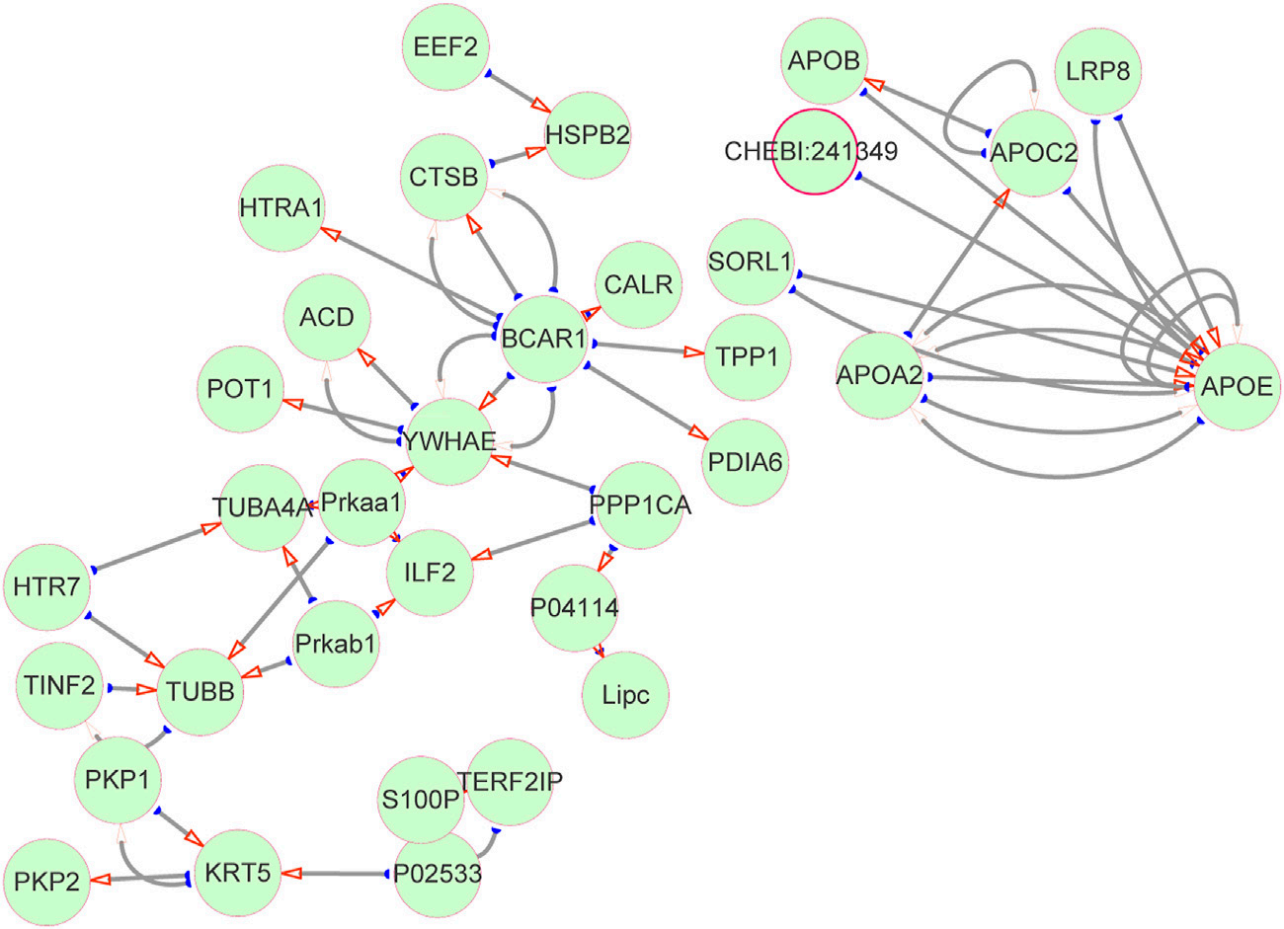
Protein–protein interaction network. DEPs interaction network diagram. The green circles represent proteins, and the red arrows point to target proteins (target proteins of DEPs), and the corresponding ones are source proteins (DEPs).

Subsequently, we interrogated the interaction relationship of these DEPs. The UNIPROT_ACCESSION of DEPs was input in Cytoscape, the data were retrieved from the HPIDB database to plot the protein interaction network, and the results as shown in Figure 5. APOE is not only a target of itself but also a target protein of other APO family members. TPP1, which is significantly downregulated in MMA patients, is mainly a target protein of BCAR1, while the downregulated protein of TUBB interacts with multiple DEPs at the same time. Interestingly, both APO and TPP1 have been reported to be involved in lipid metabolism (Ghosh et al., 2017). It follows that there is a tight interaction relationship between the DEPs, which may co-act together on MMA progression, and this contribution may be related to lipid metabolism.

### C-reactive protein was positively correlated with methylmalonic acidemia

**FIGURE 6:**
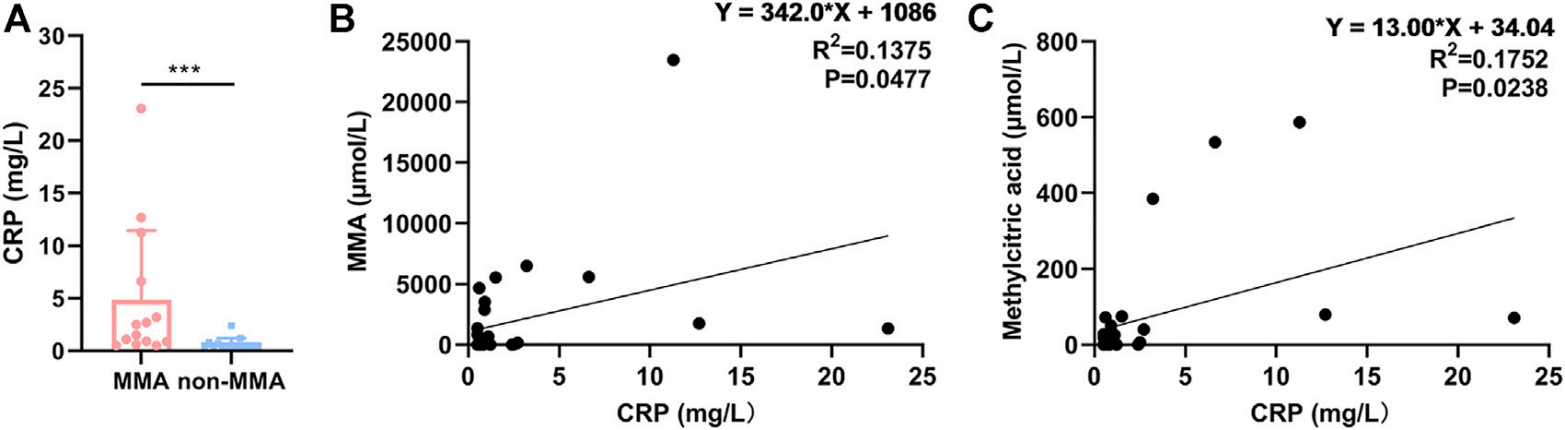
CRP was positively correlated with MMA. **(A)** Levels of CRP in serum of 14 MMA patients and 15 non-MMA (uninfected) patients. **(B)** Correlation analysis of serum CRP and MMA. **(C)** Correlation analysis of serum CRP and methylcitric acid.

To explore the reliability of the proteomic data, we expanded the sample size. We compared the levels of CRP in the serum of 14 MMA patients and 15 non-MMA (uninfected) patients, these values were in a range (MMA: 0.5–23.09; non-MMA: 0.5–2.41) consistent with the finding in serum levels of CRP (MMA: < 0.5, 0.91; no-MMA: < 0.5, 0.52) in serum of Table 2 and Supplementary Table S3. The results showed that CRP levels were significantly up-regulated in MMA patients compared with those of non-MMA patients (Figure 6A). Further analysis showed that serum CRP levels were significantly positively correlated with MMA levels and methylcitric acid levels in MMA patients (Figures 6B,C). In fact, we have also analyzed the correlation between CRP content and patient age, gender, C3, C0, and other indicators, but no correlation was found. Together, these results support our proteomic data and suggest a positive correlation between CRP and MMA. The CRP validation is consistent with clinical metadata shown for the patients and thus gives confidence to the iTRAQ dataset as a whole.

## Discussion

Isolated MMA is an inborn error of metabolism caused by the impaired isomerization of L-MCM to succinyl-CoA mutase. Normally, methylmalonic acid is the product of odd-chain fatty acids, some branched-chain amino acids, and cholesterol *via* the catabolic pathway of the propionyl coenzyme A to Krebs cycle (Richard et al., 2006). *MUT* gene mutations prevent the proper breakdown of these molecules, leading to the accumulation of a large number of compounds in the tissue that eventually form the pathologic characteristics of MMA. Considering Since the fact that the clinical diagnosis of MMA relies on metabolite detection, at present, the alteration of the metabolome caused by mutations in the *MUT* gene has been greatly recognized. In this study, we characterized the protein expression profiles of three isolated MMA patients and three healthy controls by iTRAQ technology in an attempt to connect the relationship between *MUT* mutations and abnormal protein expression at the global level in MMA patients.

In our study, a total of 94 DEPs were identified between MMA patients and healthy control, including 58 up-regulated and 36 down-regulated DEPs in MMA patients. Among them, compared with the control group, the most significantly up-regulated proteins were CRP and immunoglobulins, and the top five most significantly down-regulated proteins were all different types of immunoglobulins. Immunoglobulins appear repeatedly in the differential protein expression profiles of MMA patients and healthy control, which emphasizes that the immune environment may be strongly altered in MMA patients. This is consistent with the clinical outcome of MMA patients. Wong et al. (1992) reported that the population of B-lymphocytes was aggressively diminished and the ratio of CD4/CD8 was reversed in MMA infants which were regarded as having severe immunodeficiency. Altun et al. (2021) also proved that immunoglobulins and various immune cell types (including memory B cells, recent thymic emigrants, and Naïve helper T cells) exhibit marked defects. Importantly, intravenous administration of immunoglobulin has a dramatic rescue effect on the adverse clinical manifestations of MMA (Aikoh et al., 1997). Abnormal metabolism of MMA is located in mitochondria and leads to the failure of the citric acid cycle and affects the mitochondria function (Forny et al., 2021). It is reported that mitochondrial function determines individual B-cell fates, as well as their immunoglobulins (Jang et al., 2015). Therefore, immunoglobulins may be a potential idea to reverse-interrogate the consequences of *MUT* mutations in MMA patients.

In addition, CRP is massively elevated in the plasma of patients with MMA, and we speculate that there may be a point of association. It is generally agreed that CRP is highly conserved and is part of innate immune function, and occurs locally in inflamed or damaged tissues. As part of inflammation, elevated CRP is associated with cardiovascular disease, which is well-established by the Centers for Disease Control and the American Heart (Black et al., 2004). Wang et al. (2020) demonstrated that cardiovascular disease was strongly associated with mitochondria-derived MMA. Thus, the inflammatory response may be responsible for the accumulation of CRP protein in MMA serum. However, Del Giudice and Gangestad, (2018) proposed a new theory that CRP may exist in the absence of inflammation and have a net anti-inflammatory effect. In this case, elevated CRP indicates that the body is investing in protecting, preserving, and/or repairing somatic tissue. Depending on the state of the organism, maintenance may translate into responses including inflammation or tolerance. We conjecture that the currently recognized function of CRP may be only the tip of the iceberg and that the increase of CRP in MMA patients may be either a mechanism of clearance of damaged mitochondria or an unknown one. Collectively, CRP is one of the ways to understand the pathological mechanism of MMA.

Moreover, we found significant enrichment of DEPs in cholesterol metabolism pathways and lipid-related diseases. Similar results were found in zebrafish, where cobalamin C (cblC) deficiency caused by mutations in MMA resulted in deregulated expression of genes involved in cholesterol metabolism as demonstrated by Sloan et al. (2020). This can be explained by the physiological function of MCM enzymes. The MCM enzyme is encoded by the *MUT* gene and is also required for the degradation of a variety of amino acids, odd-chain fatty acids, and cholesterol (Keyfi et al., 2016). Thus, MMA patients with *MUT* mutations have abnormal cholesterol metabolic pathways. In addition, Manoli and Venditti (2005) reviewed that the major secondary complications of MMA include mental retardation, metabolic stroke, progressive impairment of renal function, pancreatitis, acrodermatitis-enteropathica-like lesions, and functional immune impairment. Interestingly, the occurrence of these diseases is associated with cholesterol or lipid abnormalities. For example, studies have shown that abnormal mutations in cholesterol biosynthetic genes are a possible cause of intellectual disability (Besnard et al., 2019). Mental retardation is also accompanied by a significant accumulation of lipids (Elmahgoub et al., 2009). The total cholesterol levels and incidence of severe acute pancreatitis present a U-shaped relationship (Hong et al., 2020). Therefore, the disease predicted by DEPs may be similar to the complications of MMA, with the common denominator of abnormal cholesterol or lipid metabolism. Summarily, cholesterol metabolism may be one of the important bridges between communicating MUT gene mutations and MMA disease.

The CRP validation is consistent with clinical metadata shown for the patients and thus gives confidence to the iTRAQ dataset as a whole. However, the clinical validation of only one DEP also limits the generalizability of the results of this study to a certain extent. Therefore, in the further continuation, we will continue to explore the expression patterns and functions of four DEPs, including two upregulated DEPs (CTSC and SAR1A) and two downregulated DEPs (lactotransferrin and CETP) in MMA patients compared with healthy control. As mentioned in the results, DEPs associated with lipid metabolic diseases, which prompted us to examine the relationship between lipid-related DEPs and MMA progression. Therefore, CTSC (Rufinatscha et al., 2017), SAR1A (Sane et al., 2019), lactotransferrin (Kovacic et al., 2014), and CETP (Trinder et al., 2021) were selected for future validation due to their significant *p*-value and their correlation with lipid metabolism. We expect to provide more theoretical bases and evidence for clinical determination.

In conclusion, the present study characterized the protein expression profile of isolated MMA patients with *MUT* mutation and identified DEPs compared with healthy controls. These DEPs were mainly enriched in GO entries associated with an immune function such as neutrophil-mediated immunity, neutrophil activation, neutrophil degranulation, neutrophil activation involved in immune response, and membrane protein function. KEGG revealed that these DEPs were mainly enriched in lysosome and cholesterol metabolism pathways. Moreover, these DEPs contributed to lipid metabolic diseases. The DEPs and their pathways may be useful targets for new therapies to alleviate disease symptoms, importantly, candidate DEPs, such as CRP and immunoglobulins, and are associated with specific pathways were can be measured by routine tests (already available) and thus implemented in a clinical setting.

## Data Availability

The data presented in this study are deposited in the repository, accession number: PXD034075 (PRIDE Archive, https://www.ebi.ac.uk/pride/archive). The other data found in the article/Supplementary Material.
