# Supplementary material for: Serum differential proteomic profiling of patients with isolated methylmalonic acidemia by iTRAQ
Source: Front Genet. 2022 Aug 29;13:765637. doi: 10.3389/fgene.2022.765637 (PMC9464863; doi:10.3389/fgene.2022.765637)
Supplement: Supplementary file 2 [file Table1.DOCX]

Supplemental Table 1. Clinical information on samples for validation of differentially expressed proteins.

| Group | Sample | Sex | Age  (Months) | CRP  (mg/L) | MMA  (mmol/mol creatinine) | Methylcitric acid  (mmol/mol creatinine) | C0 (μmol/L) | C3 (μmol/L) | C3/C0 | C3/C2 |
| --- | --- | --- | --- | --- | --- | --- | --- | --- | --- | --- |
| MMA | 1 | F | 3 | 0.89 | 2883.0 | 37.09 | 27.95 | 48.6 | 1.739 | 2.76 |
|  | 2 | F | 24 | 0.61 | 4686.3 | 72.39 | 16.46 | 6.86 | 0.42 | 0.39 |
|  | 3 | M | 0.26 | 0.5 | 843.0 | 18.75 | 26.54 | 8.89 | 0.33 | 0.46 |
|  | 4 | M | 0.13 | 11.28 | 23492.4 | 587.30 | 14.94 | 10.92 | 0.73 | 0.61 |
|  | 5 | M | 5.6 | 0.5 | 1373.8 | 27.76 | 11.03 | 8.13 | 0.61 | 0.56 |
|  | 6 | M | 1.1 | 3.22 | 6501.5 | 384.68 | 2.81 | 2.11 | 0.75 | 0.91 |
|  | 7 | M | 0.2 | 6.63 | 5592.2 | 534.17 | 14.6 | 8.63 | 0.59 | 0.42 |
|  | 8 | M | 1 | 1.5 | 5555.3 | 75.19 | 54.2 | 6.14 | 0.11 | 0.16 |
|  | 9 | M | 36 | 2.5 | 37.5 | 6.34 | 48.76 | 7.96 | 0.16 | 0.23 |
|  | 10 | F | 2.4 | 1.1 | 676.5 | 25.93 | 13.82 | 3.42 | 0.25 | 0.33 |
|  | 11 | M | 2 | 12.7 | 1758.4 | 79.60 | 9.51 | 2.37 | 0.25 | 0.54 |
|  | 12 | M | 2 | 23.09 | 1340.4 | 71.11 | 12.16 | 2.9 | 0.24 | 0.55 |
|  | 13 | M | 16 | 0.91 | 3526.3 | 50.61 | 66.87 | 23.11 | 0.35 | 0.68 |
|  | 14 | M | 4.3 | 2.72 | 153.2 | 40.41 | 32.56 | 24.65 | 0.76 | 0.91 |
| non-MMA | 1 | F | 0.2 | 0.5 | 0.0 | 0.0 | 24.55 | 1.57 | 0.06 | 0.07 |
|  | 2 | M | 0.26 | 0.5 | 0.0 | 0.0 | 26.52 | 2.4 | 0.09 | 0.12 |
|  | 3 | F | 0.26 | 0.5 | 0.0 | 0.0 | 21.02 | 2.43 | 0.12 | 0.14 |
|  | 4 | M | 0.2 | 0.5 | 0.0 | 0.0 | 23.05 | 1.87 | 0.08 | 0.1 |
|  | 5 | M | 0.2 | 0.5 | 0.0 | 0.0 | 29.18 | 3.11 | 0.11 | 0.12 |
|  | 6 | M | 23 | 0.5 | 0.0 | 0.0 | 13.24 | 1.69 | 0.13 | 0.12 |
|  | 7 | F | 9 | 0.68 | 0.0 | 0.0 | 24.66 | 1.84 | 0.07 | 0.08 |
|  | 8 | M | 2 | 0.5 | 0.0 | 0.0 | 31.57 | 2.64 | 0.08 | 0.12 |
|  | 9 | F | 16 | 0.5 | 0.0 | 0.0 | 18.08 | 2.05 | 0.11 | 0.09 |
|  | 10 | M | 23 | 0.5 | 0.0 | 0.0 | 21.16 | 1.61 | 0.08 | 0.08 |
|  | 11 | M | 1.7 | 1.21 | 0.0 | 0.0 | 25.98 | 1.94 | 0.07 | 0.08 |
|  | 12 | F | 17 | 2.41 | 0.0 | 0.0 | 30.15 | 1.83 | 0.06 | 0.17 |
|  | 13 | M | 19 | 0.5 | 0.0 | 0.0 | 38.64 | 2.09 | 0.05 | 0.05 |
|  | 14 | M | 12 | 0.82 | 0.0 | 0.0 | 9.59 | 0.97 | 0.1 | 0.08 |
|  | 15 | M | 22 | 0.5 | 0.0 | 0.0 | 20.14 | 2.07 | 0.1 | 0.07 |

Note: “F” means female, “M” means male. For the index CRP, clinically, all values below 0.5 are recorded as “< 0.5”, here, for the convenience of statistics, all of them are recorded as 0.5. For the non-MMA group, the samples were obtained from the biochemical test results of children diagnosed with non-MMA diseases. The clinical diseases of non-MMA samples were as follows: 1: Low birth weight infant, 2: Premature infant, 3: Severe neonatal asphyxia (post-resuscitation sample), 4: Premature infant, 5: Premature infant, 6: Colon flexure, 7: Archostegnosis, 8: Archostegnosis, 9: Colon flexure, 10: Left cryptorchidism, 11: Archostegnosis, 12: Sigmoid colon flexure, 13: Sigmoid colon juvenile polyp, 14: Left inguinal hernia, 15: Colon hypertrophy.
