# Supplementary material for: Serum differential proteomic profiling of patients with isolated methylmalonic acidemia by iTRAQ
Source: Front Genet. 2022 Aug 29;13:765637. doi: 10.3389/fgene.2022.765637 (PMC9464863; doi:10.3389/fgene.2022.765637)
Supplement: Supplementary file 3 [file Table3.DOC]

Supplemental Table 3. The CRP levels of no-MMA patients prior to sampling.

|  | Sample 004 | Sample 005 | Sample 006 |
| --- | --- | --- | --- |
| CRP (mg/L) | 0.52 | ＜0.5 | ＜0.5 |
