# Supplementary figures and images for: Serum differential proteomic profiling of patients with isolated methylmalonic acidemia by iTRAQ
Source: Front Genet. 2022 Aug 29;13:765637. doi: 10.3389/fgene.2022.765637 (PMC9464863; doi:10.3389/fgene.2022.765637)

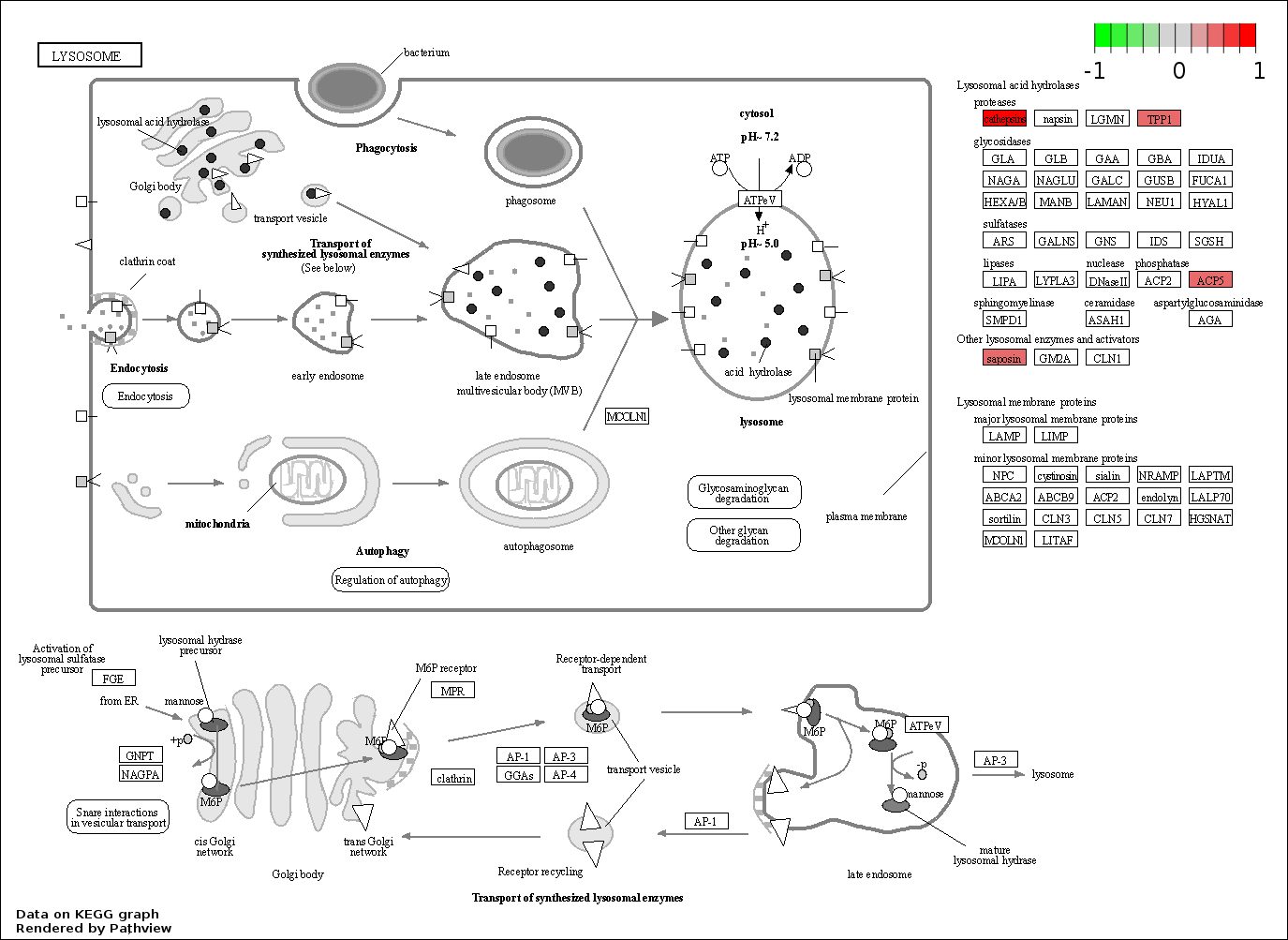

Supplement: Supplementary file 4 [file DataSheet1.ZIP › Supplemental document 1/hsa04142.pathview.png]

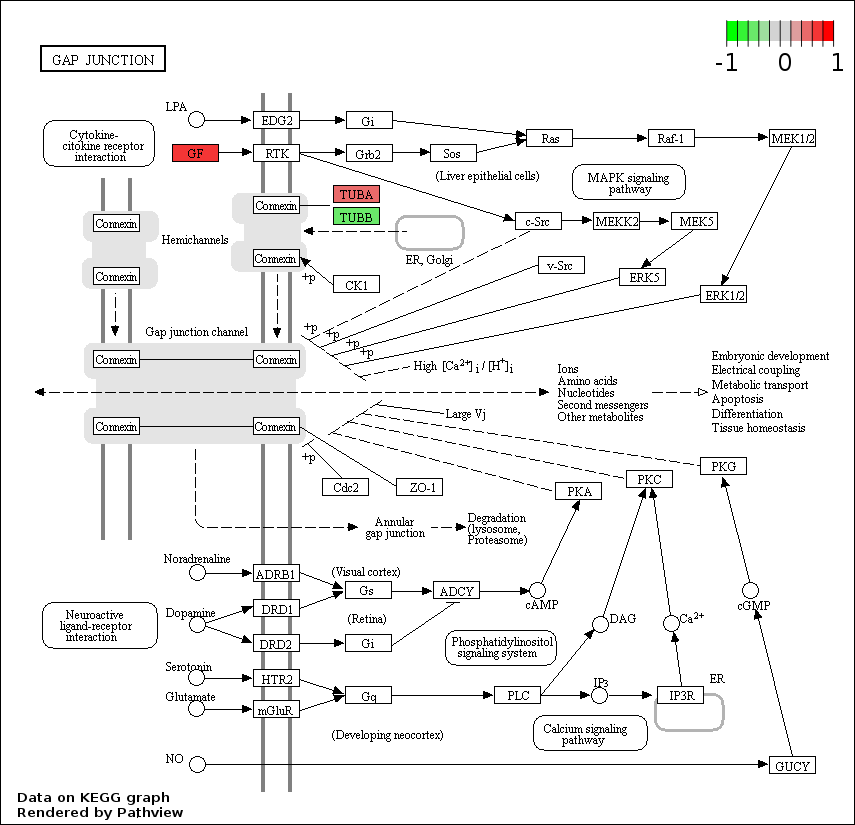

Supplement: Supplementary file 4 [file DataSheet1.ZIP › Supplemental document 1/hsa04540.pathview.png]

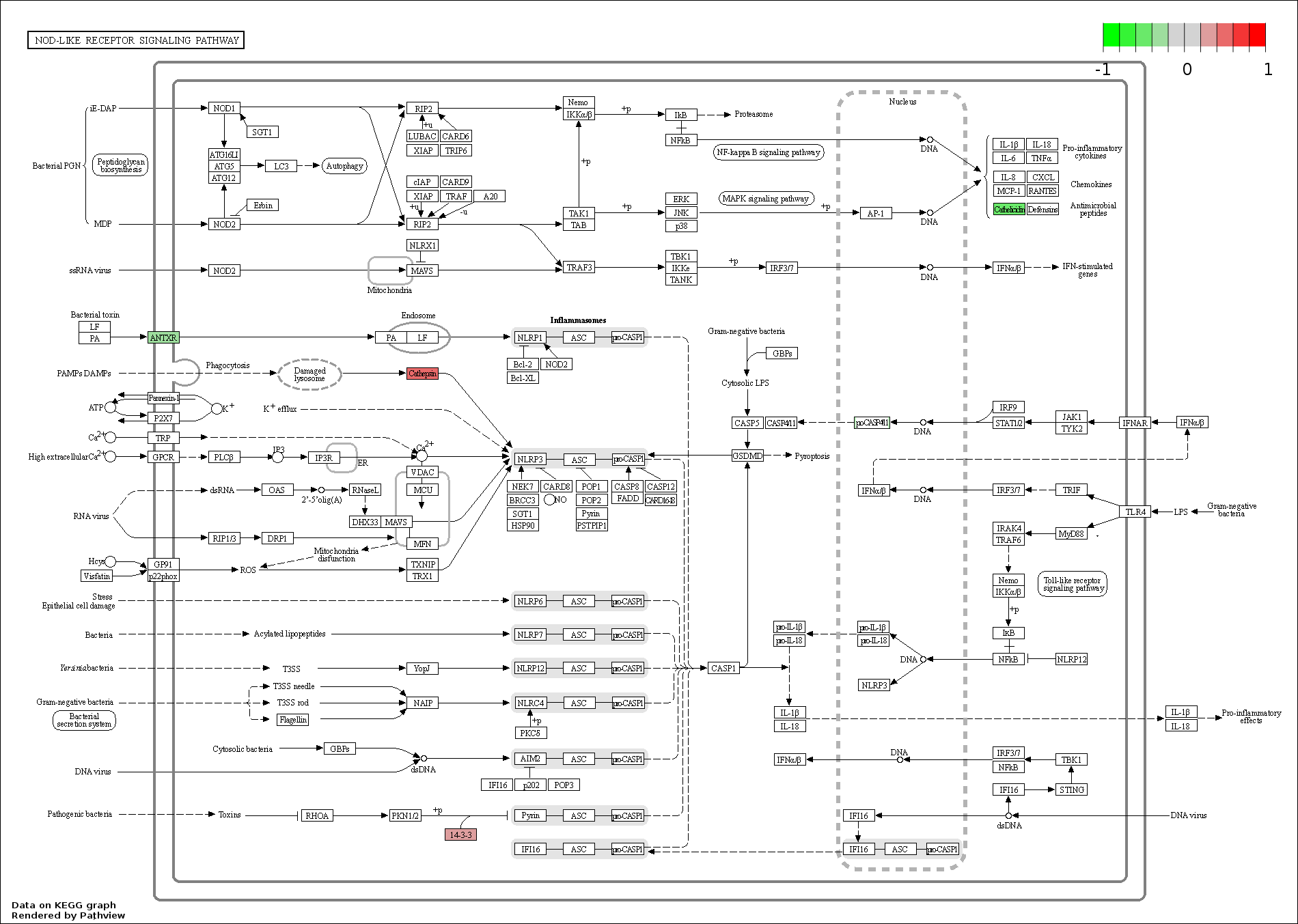

Supplement: Supplementary file 4 [file DataSheet1.ZIP › Supplemental document 1/hsa04621.pathview.png]

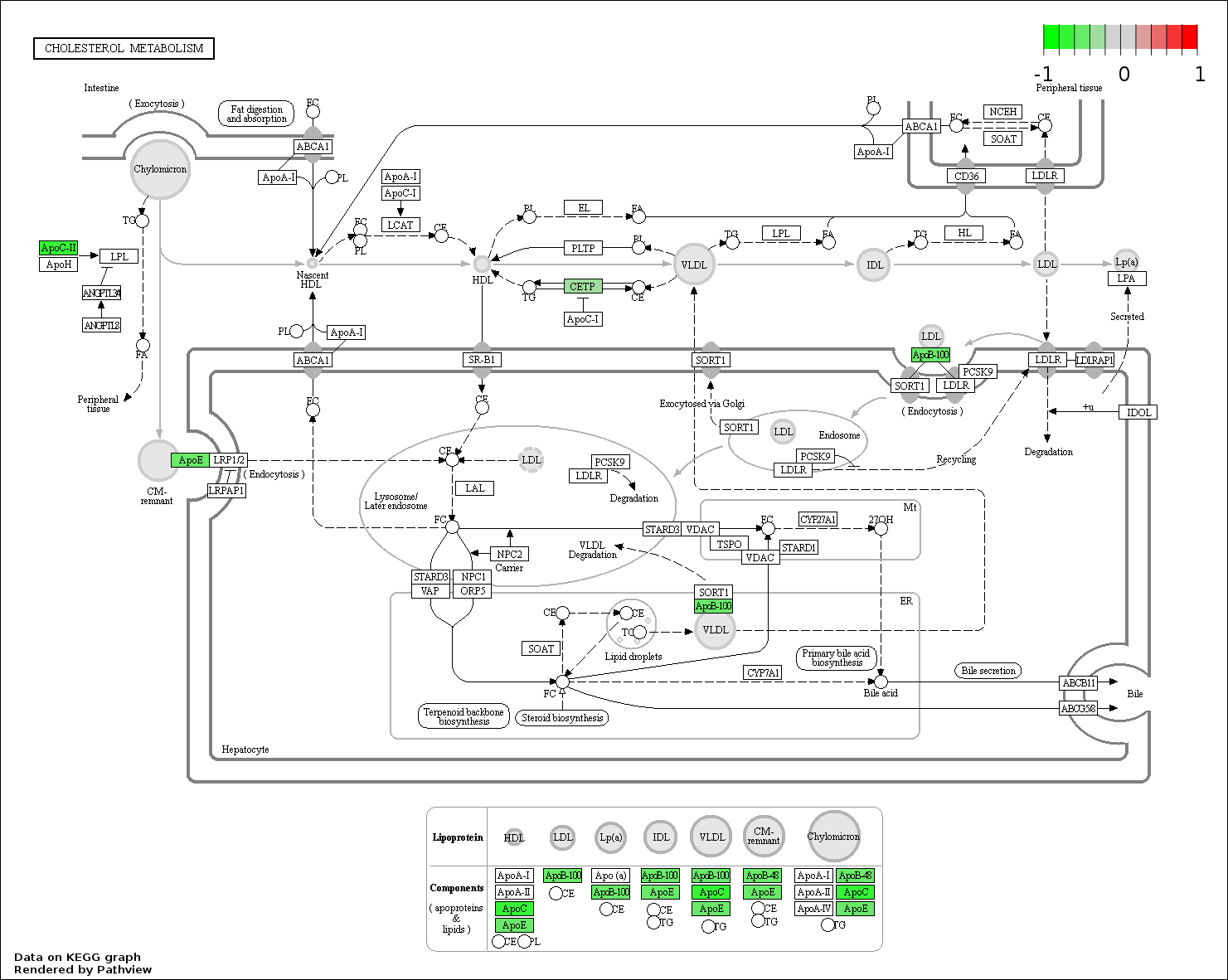

Supplement: Supplementary file 4 [file DataSheet1.ZIP › Supplemental document 1/hsa04979.pathview.png]
